# A prognostic inflammation-nutrition reserve index for 180-day functional-survival outcomes after acute ischemic stroke in patients aged 80 years or older

**DOI:** 10.3389/fneur.2026.1910335

**Published:** 2026-10-23

**Authors:** Lianhong Zhang, Jinghua Peng

**Affiliations:** Department of Neurology, Jiujiang First People's Hospital, Jiujiang, Jiangxi, China

**Keywords:** acute ischemic stroke, inflammation, MRS, nutrition, oldest-old, prognosis

## Abstract

**Background:**

Patients aged 80 years or older with acute ischemic stroke (AIS) have heterogeneous functional-survival outcomes despite similar clinical severity. We evaluated whether a routine laboratory-derived Prognostic Inflammation-Nutrition Reserve Index (PINRI) was associated with the 180-day ordinal functional survival state.

**Methods:**

This single-center cohort study screened consecutive patients admitted with suspected AIS from January 2021 through December 2024. Admission PINRI combined an internally standardized logarithmic C-reactive protein-to-albumin ratio, the logarithmic systemic inflammatory response index, and the inverse of the prognostic nutritional index; no transportable clinical threshold was proposed. The primary outcome was 180-day modified Rankin Scale (mRS) 0–2, mRS 3–5, or death.

**Results:**

Among 278 analyzable patients, 111 (39.9%) achieved mRS 0–2, 114 (41.0%) had mRS 3–5, and 53 (19.1%) died; 167 (60.1%) experienced dependency or death. Each 1-SD increase in admission PINRI was associated with a worse functional survival state after adjusting for selected clinical covariates (adjusted common odds ratio [aOR], 1.98; 95% CI, 1.48–2.65; *p* < 0.001). The aOR for the highest versus lowest tertile was 3.43 (95% CI, 1.74–6.74; *p* < 0.001). The first-week PINRI burden remained associated with a worse outcome among 241 day-7 landmark survivors (aOR, 1.80; 95% CI, 1.19–2.72; *p* = 0.005). Adding PINRI improved the internally optimism-corrected C-statistic for dependency/death from 0.810 to 0.834 (difference, 0.024; *p* = 0.033). Comparator single-marker C-statistics were 0.821–0.825; none differed significantly from PINRI after false discovery rate (FDR) control.

**Conclusion:**

Higher admission PINRI and first-week PINRI burden were associated with a worse 180-day functional survival state and provided modest incremental information beyond the clinical model. Multicenter validation and local calibration are required before implementation.

## Introduction

Acute ischemic stroke (AIS) remains a leading cause of death and disability worldwide, with older adults bearing an increasingly disproportionate share of its clinical burden as populations age (1, 2). Current clinical guidance emphasizes rapid diagnosis, early treatment, complication prevention, secondary prevention, and rehabilitation pathways for ischemic cerebrovascular disease (3, 4). This need is especially pronounced among patients aged 80 years or older, for whom premorbid disability, frailty, comorbidity, dysphagia, infection, and limited physiological reserve may materially influence outcomes alongside the index neurological deficit (5, 6). In a recent cohort of AIS patients aged 80 years or older after intravenous thrombolysis, baseline NIHSS and admission glucose were independent predictors of poor prognosis, illustrating the importance of admission severity and metabolic reserve in this age group (7).

Acute cerebral ischemia triggers peripheral immune activation, leukocyte redistribution, endothelial injury, catabolism, and subsequent immune dysregulation that may influence infections, tissue repair, and rehabilitation tolerance (8). Poststroke immunodepression and infection are clinically important because pneumonia and other infections cluster among more severe and dysphagic patients and may worsen functional trajectories even when they partly reflect baseline severity (9). In parallel, nutritional risk is common and clinically relevant in neurological inpatients, particularly when dysphagia, catabolism, and inflammation coexist (6, 10). In older adults with acute ischemic stroke, nutritional depletion has been associated with poor functional outcomes, highlighting nutrition-related vulnerability as an important component of prognostic assessment (11).

Routine blood tests provide a practical way to capture these systemic domains without adding imaging, omics, wearable monitoring, or costly assays. C-reactive protein-to-albumin ratio (CAR) combines inflammatory activation with albumin-related nutritional and inflammatory reserve; recent stroke meta-analyses in stroke populations support its adverse prognostic association, and thrombolysis cohorts have linked higher ratios to poor functional outcomes (12, 13). Leukocyte-derived indices, such as the systemic immune-inflammation index (SII) and the systemic inflammatory response index (SIRI), integrate neutrophils, monocytes, lymphocytes, and platelet counts, and systematic reviews suggest that higher values are associated with unfavorable outcomes after acute ischemic stroke (14, 15). Albumin and lymphocyte-based measures, including the prognostic nutritional index (PNI), also have plausible relevance to stroke outcome (11). The modified Rankin Scale (mRS) is widely used and has substantial validity in stroke outcome assessment (16). Ordinal mRS analyses can preserve information that is lost when outcomes are dichotomized, particularly in populations where complete independence is less common, and transitions between moderate and severe dependency remain meaningful for patients and families (17). We therefore conducted a single-center derivation cohort study to evaluate the prespecified Prognostic Inflammation–Nutrition Reserve Index (PINRI), derived from routine admission measurements of the C-reactive protein-to-albumin ratio, systemic inflammatory response index, and inverse prognostic nutritional index. We hypothesized that higher admission PINRI would be associated with worse 180-day ordinal functional survival state in AIS patients aged 80 years or older and that adding PINRI to a parsimonious clinical model would provide a modest incremental prognostic value.

## Methods

### Study design and setting

This non-interventional, single-center cohort study was conducted at Jiujiang First People’s Hospital. Consecutive adults admitted with suspected AIS between 1 January 2021 and 31 December 2024 were identified and screened using electronic medical records, laboratory information systems, nursing assessments, treatment logs, discharge summaries, outpatient records, and standardized telephone follow-up data. The follow-up database was administratively closed on 31 July 2025, after completion of the latest allowable 180-day assessment window for December 2024 admissions. Hospitalization and laboratory variables were obtained from routinely collected records, and 180-day mRS and vital status were ascertained or adjudicated through standardized follow-up when routine records were incomplete. The analysis was designed as a prognostic-factor and incremental-value study using routine numeric clinical data only; imaging predictors, omics, and wearable data were not used. Reporting followed STROBE guidance for cohort studies (18) and relevant TRIPOD reporting principles for transparent reporting of regression-based prognostic model performance (19).

This study was conducted in accordance with the Declaration of Helsinki. The study protocol, including the collection of routinely recorded clinical and laboratory data and the ascertainment and adjudication of 180-day functional and vital-status outcomes, was reviewed and approved by the Ethics Committee of Jiujiang First People’s Hospital. Data were de-identified before analysis, linkage files were stored separately, and only aggregate results were reported. Data quality procedures included consecutive screening logs, duplicate admission checks, impossible-date checks, harmonization of laboratory units, range and outlier review against source records, double extraction of a random 10% sample, and blinded mRS; blinding status was recorded for every outcome ascertainment route. Selection bias was addressed by reporting all exclusions in the flow diagram and by comparing all 26 unique final-AIS patients excluded for missing baseline components (*n* = 17) or missing 180-day outcomes (*n* = 9) with the analyzed cohort in terms of age, sex, and admission NIHSS. Reverse causation from baseline infection or fever was addressed through exclusion sensitivity analysis, while immortal time bias was addressed by using admission PINRI as the full-cohort exposure and limiting trajectory analyses to a day-7 landmark cohort. The Ethics Committee waived the requirement for written informed consent for the use of routinely collected clinical data. Verbal informed consent was obtained from patients or their legally authorized representatives before telephone follow-up, in accordance with the approved protocol.

### Participants, time zero, and eligibility

Time zero was the first registered hospital-arrival timestamp for the index AIS hospitalization, defined as emergency department arrival time for emergency admissions and direct ward admission time for direct admissions. Patients were eligible if they were aged 80 years or older at time zero, had a final clinical diagnosis of AIS consistent with clinical guidance (3, 4), were admitted during the study period, presented within 7 days of symptom onset or last-known-well time, had at least one complete baseline complete blood count and biochemical panel within 24 h after time zero, and had ascertainable 180-day vital status and/or mRS. Patients were excluded for primary intracerebral hemorrhage, subarachnoid hemorrhage, cerebral venous thrombosis, tumor, trauma, stroke mimic or final non-AIS diagnosis, in-hospital stroke after admission for another primary disease, active hematological malignancy or chemotherapy within 3 months when leukocyte counts were directly distorted, end-stage liver failure or albumin infusion before baseline albumin, missing key inflammatory-nutritional components after reasonable data recovery, or unavailable 180-day outcome. When a patient had more than one qualifying AIS admission, only the first qualifying admission was used to avoid within-person correlation.

### Exposure definition and laboratory timing

The primary exposure was admission PINRI, a predefined internally standardized composite prognostic index calculated as z[ln(CAR + 0.01)] + z[ln(SIRI + 0.01)]—z(PNI), where CAR was C-reactive protein in mg/L divided by albumin in g/L, SIRI was neutrophil count multiplied by monocyte count divided by lymphocyte count, and PNI was albumin in g/L plus 5 multiplied by lymphocyte count in 10^9^/L. The small constant in log-transformed terms was used to avoid log-zero problems after laboratory lower-limit handling. The component z-scores and the final summed composite were standardized within the analytic cohort; a higher 1-SD PINRI represented a more adverse composite inflammatory-nutritional laboratory profile. Baseline values were the first available laboratory values within 24 h after time zero and before albumin infusion, major blood product administration, or other events likely to distort baseline albumin or cell-count interpretation. For patients with repeated routine tests, PINRI was recalculated at day 3 using the closest value from 48 to 96 h and at day 7 or discharge using the closest value from day 5 to day 9 or the final value before discharge; the admission-cohort centering and scaling constants were applied to all time points to keep repeated PINRI values on the same scale. First-week PINRI burden was computed as the trapezoidal area under the PINRI-time curve divided by the number of observed days, but this dynamic exposure was analyzed only in a day-7 landmark cohort of patients alive at day 7 with repeated laboratory measurements.

The component set, directionality, transformations, and equal-weight formula were recorded in a dated statistical analysis plan before PINRI variables were linked to 180-day outcomes and before outcome-association or model-performance analyses. CAR was selected to represent CRP relative to albumin, SIRI to represent neutrophil-monocyte predominance relative to lymphocytes, and PNI to represent albumin and lymphocyte-based nutritional/immune reserve; all three use routine admission tests and have stroke-specific prognostic support (12–15). Equal weights were used after z standardization to avoid outcome-derived coefficient weighting and associated overfitting in this single-center derivation cohort. CAR and SIRI were log-transformed because their positive ratio/product distributions were right-skewed, whereas PNI was retained on its approximately symmetric original scale. The cohort-specific centering and scaling constants and the complete reproduction formula are provided in Supplementary Table S1.

### Outcomes and covariates

The primary outcome was 180-day functional survival state, defined as a three-level ordinal endpoint: mRS 0–2 indicating functional independence, mRS 3–5 indicating dependency among survivors, and mRS 6/death indicating death. The target assessment date was 180 days after time zero, with an allowable window from 166 to 194 days. Death before 180 days was assigned mRS 6. Secondary outcomes included dependency/death (mRS 3–6), severe dependency/death (mRS 4–6), and all-cause death by 180 days; discharge mRS, in-hospital pneumonia or urinary tract infection, length of stay, recurrent stroke, and all-cause readmission were described only when reliably recorded. Outcome source was assigned hierarchically as routine clinic assessment, standardized telephone assessment performed within the target window, mRS documented in a medical-record follow-up encounter within the window, or retrospective two-reviewer adjudication when no contemporaneous mRS was available. PINRI was calculated only after outcome values were assigned and locked; research telephone assessors and adjudicators were not shown the calculated PINRI or its component biomarker values. Stroke severity was measured by NIHSS (20). Baseline infection or fever was defined based on admission documentation of suspected or confirmed infection, antimicrobial treatment for infection, or temperature ≥38.0 °C before or at the time of baseline blood sampling. Dysphagia screen positivity referred to the routine admission bedside swallowing screen recorded before oral intake for all 278 analyzed patients. The primary adjustment set was selected before analysis and included age, sex, prestroke mRS, admission NIHSS, atrial fibrillation, baseline estimated glomerular filtration rate, admission glucose, and reperfusion therapy coded as intravenous thrombolysis and/or endovascular therapy. Onset-to-door time, diabetes mellitus, baseline infection/fever, and dysphagia were described and considered in sensitivity interpretation but were not added simultaneously to the primary model because of parameter burden and their potential roles as severity markers, mediators, or colliders. Post-baseline pneumonia, nutritional intervention, ICU transfer, length of stay, and discharge mRS were not included in the primary model because they could lie on the prognostic pathway.

For admissions whose assessment window had closed before prospective follow-up began, two trained reviewers independently assigned mRS from the nearest date-anchored outpatient, rehabilitation, readmission, and activities-of-daily-living documentation surrounding day 180, using predefined mRS rules; disagreements were resolved by consensus, and later records were not used to carry functional status backward. Cases without sufficient date-anchored information to assign the 180-day outcome were excluded. The three-level primary endpoint was selected *a priori* to distinguish functional independence (mRS 0–2), dependency among survivors (mRS 3–5), and death, preserving the clinically consequential separation of death from severe disability while limiting sparse-category instability in this oldest-old cohort.

### Missing data, sample size, and power rationale

Missingness was summarized for each candidate variable and by outcome-availability status before modeling. The primary outcome was not imputed. Variables with less than 5% missingness were handled using available complete values; variables with greater missingness were handled, when included in sensitivity models, using multiple imputation by chained equations with 20 imputations under a missing-at-random assumption, incorporating exposure, outcome indicator, covariates, auxiliary laboratory variables, and care-process variables, while not imputing the primary outcome for the main analysis (21). A complete-case analysis was retained as a sensitivity analysis. The analytic cohort contained 278 patients, 167 dependency/death events, 119 severe dependency/death events, and 53 deaths. This event structure supported the prespecified primary ordinal prognostic-factor analysis of PINRI with a parsimonious core clinical adjustment set. Mortality models, interaction analyses, and nonlinear visualizations were designated exploratory, while external validation was considered a separate confirmatory step. This approach is consistent with modern sample-size guidance that emphasizes model purpose, expected overfitting, and parameter burden rather than simple events-per-variable rules alone (22).

### Statistical analysis

Continuous variables were summarized as mean (standard deviation, SD) or median (interquartile range, IQR), and categorical variables were summarized as *n* (%). Baseline characteristics and unadjusted 180-day outcome distribution were described overall and by PINRI tertiles using the analysis of variance or the Kruskal–Wallis tests for continuous variables and the chi-square or Fisher exact tests for categorical variables; these comparisons were interpreted descriptively rather than as causal tests. The primary association was estimated using proportional-odds ordinal logistic regression for the three-level 180-day functional survival state, with adjusted common odds ratios reported per 1-SD increase in admission PINRI and across PINRI tertiles. The proportional-odds assumption was evaluated using score or Brant-type tests and graphical diagnostics; partial proportional-odds and multinomial models were predefined if a clinically important violation was detected. The primary model assumed a linear log-odds association for standardized PINRI. Restricted cubic splines with three knots at the 10th, 50th, and 90th percentiles were used only as exploratory visualization of continuous PINRI but not as evidence for a clinical threshold or a formal non-linear effect (23). Marginal predicted probabilities were averaged over the observed covariate distribution. Secondary logistic models evaluated mRS 3–6 and mRS 4–6. Since only 53 deaths occurred, the parsimonious death model adjusted for age, sex, prestroke mRS, admission NIHSS, and estimated glomerular filtration rate (eGFR). Incremental performance was evaluated for the base clinical model and otherwise identical extensions containing PINRI, CAR, SIRI, PNI, or SII. Internal validation used 1,000 bootstrap resamples; within each resample, the entire model was refitted, and apparent-test differences were used to correct for optimism in the C-statistic, calibration intercept, calibration slope, and Brier score. Differences in C-statistics were evaluated in paired bootstrap resamples; two-sided *p*-values were calculated from the paired-bootstrap standard error using a normal approximation, and 95% CIs were derived from the paired-bootstrap distribution. The five extension-versus-base comparisons and, separately, the four PINRI-versus-comparator comparisons were controlled using the Benjamini–Hochberg FDR procedure (24). Conventional decision curves plotted the base model, base model plus PINRI, treat-all, and treat-none strategies across threshold probabilities from 0.05 to 0.50 (25). Sensitivity analyses included the complete-case analysis, the exclusion of baseline infection or fever, binary outcome modeling, proportional-odds diagnostics with alternative model specification, if needed, and the day-7 landmark PINRI-burden analysis. Exploratory component-marker comparisons used Benjamini–Hochberg false-discovery-rate (FDR) control (24). A sensitivity proportional-odds analysis used the full seven-level mRS 0–6 scale. Analyses were performed with R version 4.3.

## Results

### Cohort flow and outcome ascertainment

Among 1,436 adult suspected AIS admissions screened from January 2021 through December 2024, 320 patients were aged 80 years or older and 1,116 were excluded because they were younger than 80 years. In total, 42 age-eligible records were excluded because of duplicate qualifying admissions, final non-AIS or stroke-mimic diagnosis, missing key baseline laboratory components, or unavailable 180-day outcome, leaving 278 analyzable patients (Figure 1). The final cohort contained 93 patients in the lowest PINRI tertile, 92 in the middle tertile, and 93 in the highest tertile. The day-7 landmark cohort included 241 patients who were alive at day 7 and had repeated laboratory data; 37 patients were not included in the landmark analysis because of death before day 7, discharge without repeat laboratory testing, or unavailable repeated laboratory data. At 180 days, 111 patients (39.9%) were functionally independent with mRS 0–2, 114 (41.0%) were alive with dependency and mRS 3–5, and 53 (19.1%) had died; therefore, 167 patients (60.1%) experienced dependency or death and 119 (42.8%) experienced severe dependency or death.

**Figure 1:**
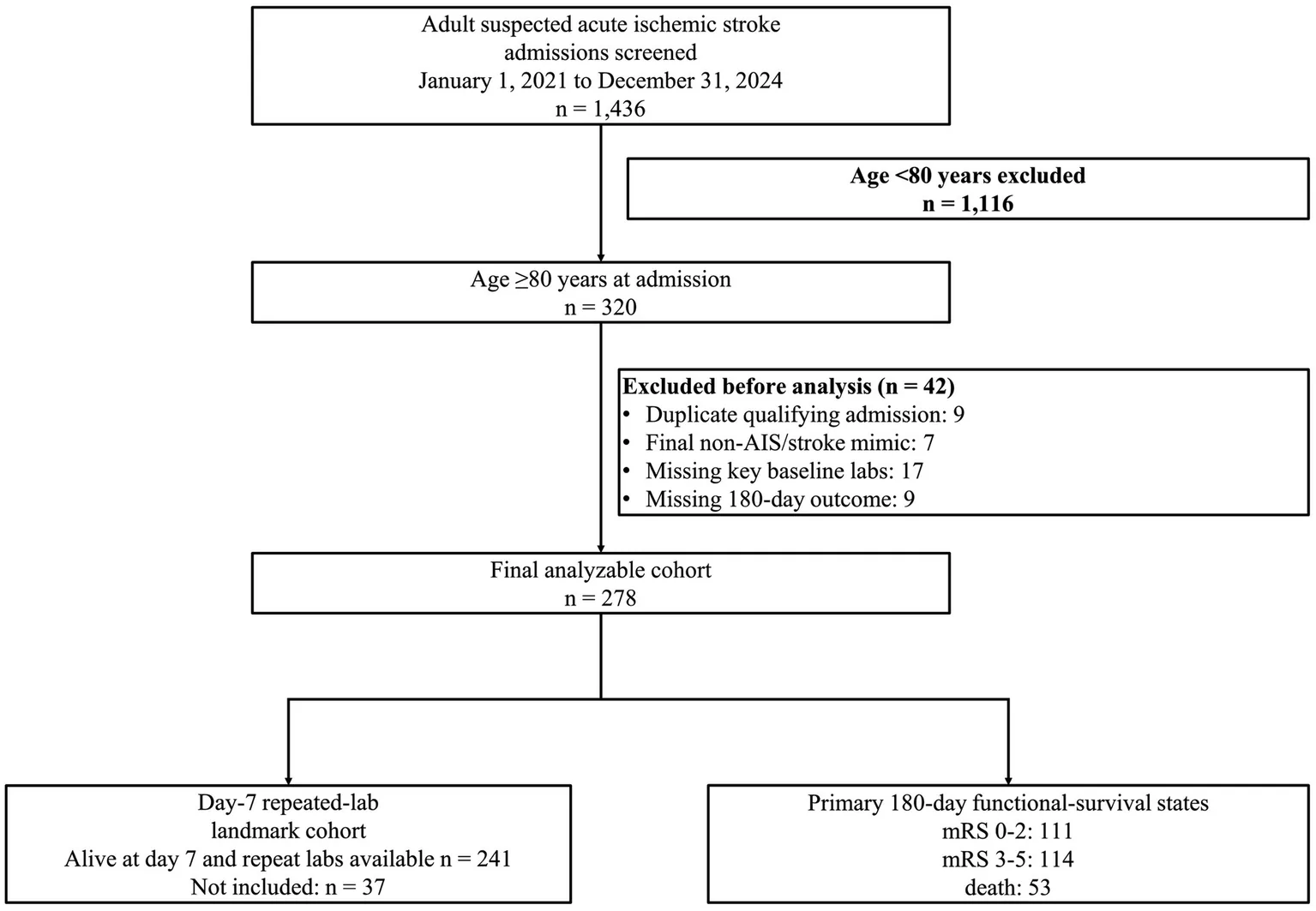
Participant flow diagram. The flow diagram shows 1,436 adult suspected AIS admissions screened, 1,116 exclusions for age younger than 80 years, 320 age-eligible patients, 42 age-eligible exclusions, 278 analyzable patients, the day-7 landmark cohort of 241 patients with repeated laboratory data, and the primary 180-day outcome states.

Outcome source was routine clinic assessment for 82 patients (29.5%), contemporaneous standardized telephone assessment for 106 (38.1%), medical-record review with an explicit mRS or vital-status entry for 66 (23.7%), and retrospective two-reviewer adjudication for 24 (8.6%). Among 225 survivors, 210 (93.3%) had mRS documented within days 166–194; the remaining 15 survivors were adjudicated from the nearest date-anchored functional records. All 53 deaths occurred by day 180 and were assigned mRS 6; thus, 263 of 278 endpoints (94.6%) were either directly assessed within the target window or established by death by day 180. Calculated PINRI was unavailable to outcome assessors for all 278 participants; for the 24 adjudicated outcomes, the adjudication form omitted PINRI and named component indices, although individual routine laboratory values in source records could not always be concealed. The 26 comparable excluded patients had a median age of 85 [82–88] years, and 13 (50.0%) were women. Among the 24 patients with a recorded score, the median NIHSS score was 7 (4–11); these distributions did not materially differ from those in the analyzed cohort (age *p* = 0.52, sex *p* = 0.86, NIHSS *p* = 0.41).

### Baseline characteristics across PINRI tertiles

The median age was 84 years [82–87] overall and did not differ across tertiles (*p* = 0.775), and 134 patients (48.2%) were women (Table 1). In contrast, admission NIHSS increased from 4 (2–7) in the lowest tertile to 8 (5–11) in the highest tertile (*p* < 0.001), and prestroke mRS was modestly higher in the highest tertile (*p* = 0.004). Baseline infection or fever was present in 32 patients (11.5%) overall but was concentrated in the highest tertile (23.7% vs. 5.4% in each lower tertile; *p* < 0.001). Dysphagia screen positivity increased from 34.4 to 62.4% across tertiles (*p* < 0.001), eGFR declined from 72.4 to 60.9 mL/min/1.73 m^2^ (*p* < 0.001), albumin declined from 41.5 to 35.9 g/L (*p* < 0.001), and CRP, CAR, SIRI, and lower PNI all worsened across tertiles. Reperfusion therapy was performed in 60 patients (21.6%) and varied by tertile (*p* = 0.039). These patterns not only supported biological plausibility but also identified important confounding and mediation concerns, particularly infection/fever, dysphagia, renal function, and stroke severity. The unadjusted outcome distribution shifted toward dependency and death across increasing PINRI tertiles (global *p* < 0.001; Figure 2). Among the 60 patients who received reperfusion therapy, 42 (15.1% of the cohort) received intravenous thrombolysis alone, 12 (4.3%) underwent endovascular thrombectomy alone, and 6 (2.2%) received both treatments.

**Table 1:** Baseline characteristics and unadjusted 180-day outcome distribution by admission PINRI tertile.

Characteristic	Overall (*n* = 278)	T1 low (*n* = 93)	T2 middle (*n* = 92)	T3 high (*n* = 93)	*p*-value
Age, years	84 [82–87]	84 [81–87]	84 [82–86]	84 [82–86]	0.775
Female sex	134 (48.2)	41 (44.1)	45 (48.9)	48 (51.6)	0.582
Admission NIHSS	6 [4–10]	4 [2–7]	6 [4–11]	8 [5–11]	<0.001
Prestroke mRS	1 [0–1]	1 [0–1]	1 [0–1]	1 [1–2]	0.004
Onset-to-door time, h	9.2 [5.1–16.3]	10.2 [5.7–15.4]	7.4 [4.8–15.3]	9.7 [5.1–17.3]	0.241
Hypertension	208 (74.8)	66 (71.0)	69 (75.0)	73 (78.5)	0.496
Diabetes mellitus	92 (33.1)	26 (28.0)	28 (30.4)	38 (40.9)	0.140
Atrial fibrillation	120 (43.2)	42 (45.2)	34 (37.0)	44 (47.3)	0.325
Prior stroke/TIA	81 (29.1)	27 (29.0)	30 (32.6)	24 (25.8)	0.595
Baseline infection/fever	32 (11.5)	5 (5.4)	5 (5.4)	22 (23.7)	<0.001
Dysphagia screen positive	144 (51.8)	32 (34.4)	54 (58.7)	58 (62.4)	<0.001
eGFR, mL/min/1.73 m^2^	65.7 (19.9)	72.4 (19.3)	63.8 (17.3)	60.9 (21.3)	<0.001
Admission glucose, mmol/L	7.1 (1.9)	6.8 (2.2)	7.0 (1.7)	7.5 (1.9)	0.037
Albumin, g/L	38.6 (3.9)	41.5 (3.3)	38.4 (3.1)	35.9 (3.3)	<0.001
Lymphocyte count, 10^9^/L	1.24 (0.36)	1.30 (0.40)	1.24 (0.35)	1.20 (0.33)	0.167
CRP, mg/L	11.4 [6.5–21.2]	7.5 [3.8–11.4]	13.1 [7.3–20.8]	16.9 [10.6–30.9]	<0.001
CAR	0.31 [0.17–0.59]	0.18 [0.09–0.27]	0.34 [0.19–0.55]	0.47 [0.30–0.84]	<0.001
SIRI	2.02 [1.33–3.14]	1.44 [0.96–1.97]	1.94 [1.39–2.93]	3.08 [2.15–4.2]	<0.001
PNI	44.8 (4.6)	47.9 (4.2)	44.6 (3.7)	41.9 (3.8)	<0.001
Reperfusion therapy	60 (21.6)	17 (18.3)	28 (30.4)	15 (16.1)	0.039
180-day outcome					<0.001*
mRS 0–2	111 (39.9)	54 (58.1)	40 (43.5)	17 (18.3)	
mRS 3–5	114 (41.0)	31 (33.3)	39 (42.4)	44 (47.3)	
Death	53 (19.1)	8 (8.6)	13 (14.1)	32 (34.4)	

Values are mean (SD), median (IQR), or *n* (%). PINRI=Prognostic inflammation-nutrition reserve index; NIHSS = National institutes of health stroke scale; mRS = modified Rankin Scale; eGFR = estimated glomerular filtration rate; CRP=C-reactive protein; CAR = C-reactive protein-to-albumin ratio; SIRI = systemic inflammatory response index; PNI = prognostic nutritional index; TIA = transient ischemic attack. PINRI tertiles were cohort-derived and are not transportable clinical thresholds. *For 180-day outcome categories, *p*-value refers to the global comparison of the three-level outcome distribution across tertiles; separate category-specific *p*-values were not interpreted.

**Figure 2:**
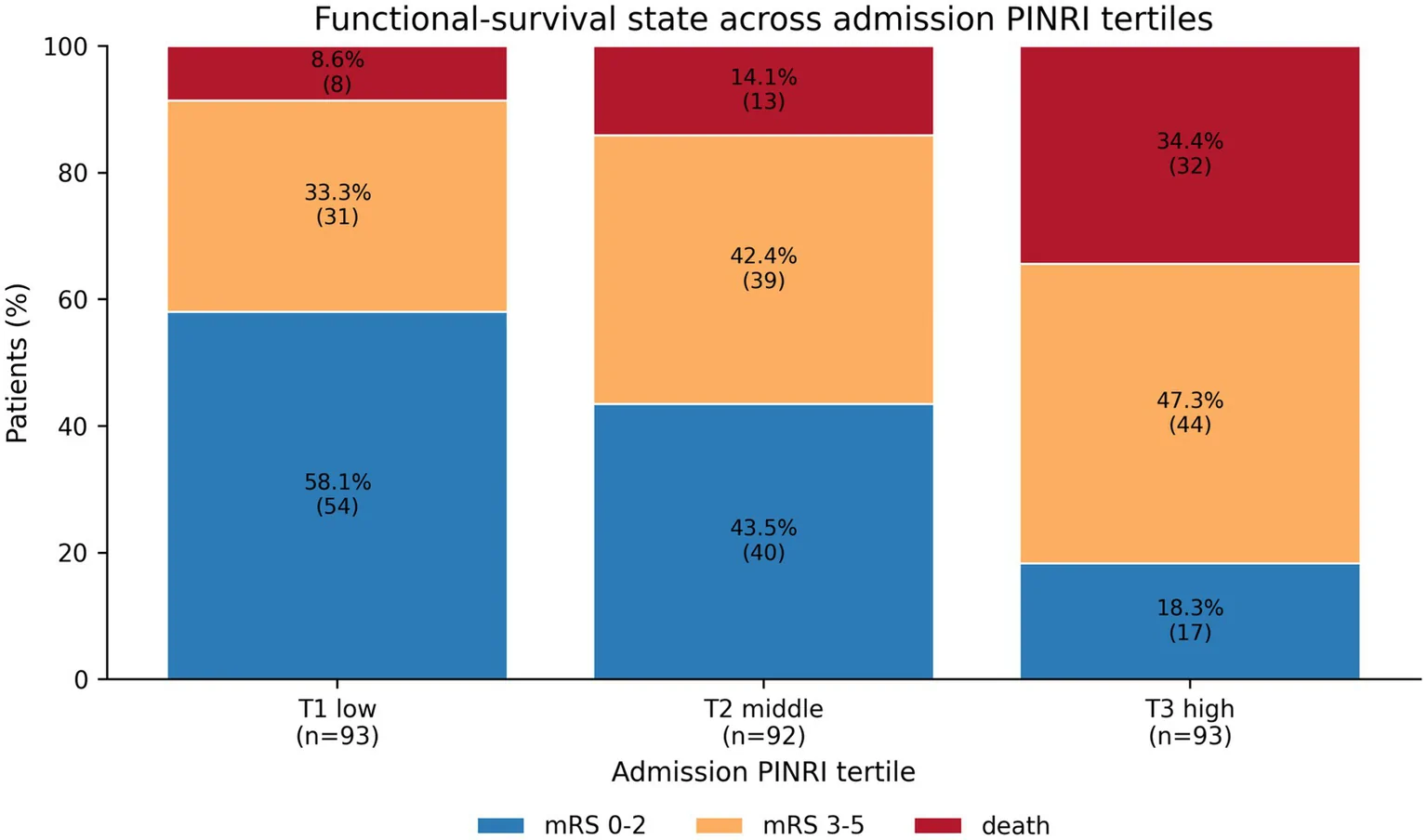
Unadjusted distribution of 180-day functional survival state across admission PINRI tertiles. Stacked bars show the unadjusted proportion and count of mRS 0–2, mRS 3–5, and death at 180 days in each cohort-specific admission PINRI tertile.

### Primary and secondary associations

The primary analysis tested whether admission PINRI remained associated with the 180-day ordinal functional-survival endpoint after adjusting for selected clinical covariates. Each 1-SD increase in admission PINRI was associated with a higher odds of worse functional survival state in the unadjusted model (common OR, 2.43; 95% CI, 1.89–3.12; *p* < 0.001) and remained significant after completely adjusting for age, sex, prestroke mRS, admission NIHSS, atrial fibrillation, eGFR, admission glucose, and reperfusion therapy (adjusted common OR, 1.98; 95% CI, 1.48–2.65; *p* < 0.001; Table 2). Compared with the lowest tertile, the middle tertile showed a non-significant increase in the odds of worse outcome (adjusted common OR, 1.61; 95% CI, 0.85–3.06; *p* = 0.148), whereas the highest tertile showed a marked increase (adjusted common OR, 3.43; 95% CI, 1.74–6.74; *p* < 0.001). Secondary binary models were directionally consistent for dependency/death (adjusted OR, 1.92; 95% CI, 1.36–2.71; *p* < 0.001), severe dependency/death (adjusted OR, 1.61; 95% CI, 1.16–2.21; *p* = 0.004), and death (parsimonious adjusted OR, 2.19; 95% CI, 1.40–3.41; *p* < 0.001). Proportional-odds diagnostics did not suggest a clinically important violation; thus, the common-odds model remained the primary model. The spline visualization showed decreasing independent survival and increasing death across the continuous PINRI range but was not used to define a threshold (Figure 3).

**Table 2:** Associations of internally standardized admission PINRI, first-week PINRI burden, and component markers with 180-day outcomes.

Analysis/exposure	Model	Effect estimate	95% CI	*p*-value	FDR q
Primary ordinal model per 1-SD admission PINRI	Unadjusted	Common OR 2.43	1.89–3.12	<0.001	—
Primary ordinal model per 1-SD admission PINRI	Age/sex/NIHSS/prestroke mRS adjusted	Common OR 2.02	1.55–2.64	<0.001	—
Primary ordinal model per 1-SD admission PINRI	Fully adjusted	Common OR 1.98	1.48–2.65	<0.001	—
Admission PINRI tertile T2 vs. T1	Fully adjusted ordinal	Common OR 1.61	0.85–3.06	0.148	—
Admission PINRI tertile T3 vs. T1	Fully adjusted ordinal	Common OR 3.43	1.74–6.74	<0.001	—
Dependency/death, mRS 3–6, per 1-SD admission PINRI	Fully adjusted logistic	OR 1.92	1.36–2.71	<0.001	—
Severe dependency/death, mRS 4–6, per 1-SD admission PINRI	Fully adjusted logistic	OR 1.61	1.16–2.21	0.004	—
180-day death, per 1-SD admission PINRI	Parsimonious adjusted logistic	OR 2.19	1.40–3.41	<0.001	—
Day-7 first-week PINRI burden, per 1-SD burden	Landmark ordinal	Common OR 1.80	1.19–2.72	0.005	—
Full mRS 0–6 shift, per 1-SD admission PINRI	Fully adjusted proportional odds	Common OR 1.84	1.42–2.39	<0.001	—
CAR, log-transformed adverse SD	Exploratory component	Common OR 1.44	1.11–1.87	0.006	0.008
SIRI, log-transformed adverse SD	Exploratory component	Common OR 1.45	1.12–1.88	0.005	0.008
SII, adverse SD	Exploratory component	Common OR 1.51	1.16–1.96	0.002	0.008
PNI, adverse SD (lower PNI)	Exploratory component	Common OR 1.48	1.13–1.93	0.005	0.008
Albumin, adverse SD (lower albumin)	Exploratory component	Common OR 1.43	1.09–1.88	0.009	0.010
CRP, log-transformed adverse SD	Exploratory component	Common OR 1.40	1.08–1.82	0.010	0.010

Fully adjusted model: age, sex, prestroke mRS, NIHSS, atrial fibrillation, eGFR, glucose, and reperfusion. Full cohort *n* = 278; day-7 landmark *n* = 241. Death model: age, sex, prestroke mRS, NIHSS, and eGFR. Components were standardized in the adverse direction, not entered with PINRI, and FDR-adjusted. SII = platelet × neutrophil/lymphocyte.

**Figure 3:**
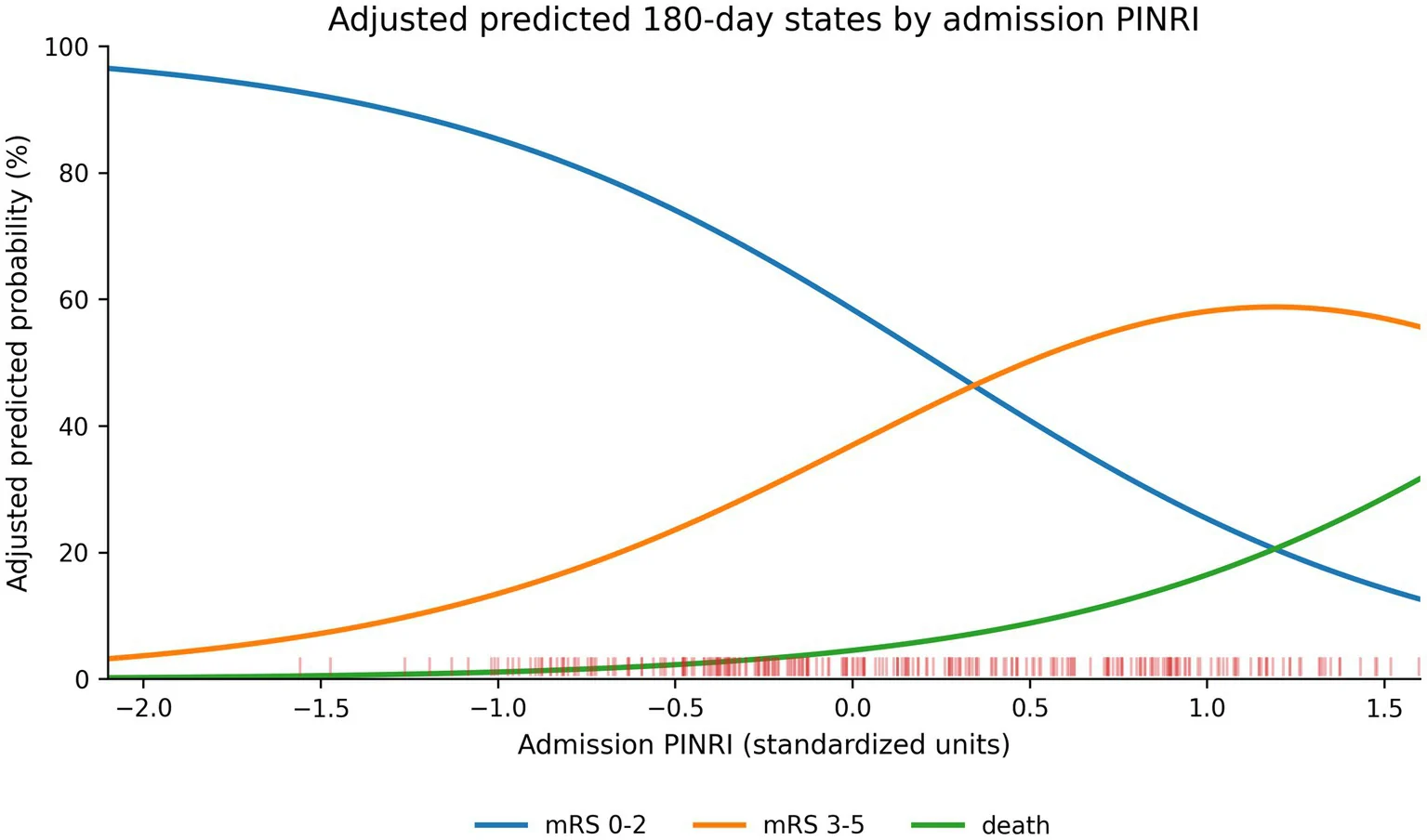
Exploratory adjusted predicted probabilities of 180-day outcomes across continuous admission PINRI. Marginal predicted probabilities were derived from the adjusted ordinal model using descriptive restricted cubic spline visualization with 3 knots for admission PINRI; the plot is not a formal test of non-linearity.

### Incremental performance, dynamic burden, and robustness

Internal model-performance analyses assessed whether PINRI improved clinically relevant stratification beyond routine predictors. Adding PINRI to the base clinical model increased the optimism-corrected C-statistic for dependency/death from 0.810 to 0.834 (difference, 0.024; bootstrap *p* = 0.033), improved the Brier score from 0.170 to 0.158, and yielded a likelihood-ratio chi-square statistic of 14.9 (*p* < 0.001), with a calibration slope of −0.02 and slope of 0.95 in the extended model (Table 3). Conventional decision curves showed only small separation of the PINRI-extended model from the base model, mainly across threshold probabilities of approximately 0.15–0.35; treat-all and treat-none strategies are shown for reference (Figure 4). In 241 day-7 landmark survivors with repeated laboratory data, each 1-SD increase in first-week PINRI burden was associated with worse 180-day functional survival state (adjusted common OR, 1.80; 95% CI, 1.19–2.72; *p* = 0.005). Sensitivity analyses were directionally consistent after excluding baseline infection or fever, in complete cases, and when using the binary dependency/death endpoint, although these rows used different analysis definitions and were interpreted as robustness checks rather than interchangeable estimates (Figure 5).

**Table 3:** Internal incremental model performance for 180-day dependency/death risk: base clinical model and single-index extensions.

Model	Optimism-corrected C-statistic	Delta C vs base (95% CI); paired *p*; FDR *q*	Calibration intercept/Slope	Brier score	Delta C vs PINRI; *p*; FDR *q*
Base clinical model	0.810	Reference	−0.01/0.96	0.170	−0.024; *p* = 0.033; *q* = −
Base clinical model + CAR	0.821	+0.011 (−0.001 to 0.023); *p* = 0.072; *q* = 0.072	−0.01/0.96	0.166	−0.013; *p* = 0.034; *q* = 0.100
Base clinical model + SIRI	0.822	+0.012 (−0.001 to 0.025); *p* = 0.070; *q* = 0.072	−0.01/0.95	0.165	−0.012; *p* = 0.050; *q* = 0.100
Base clinical model + PNI	0.824	+0.014 (0.000 to 0.028); *p* = 0.050; *q* = 0.072	0.00/0.96	0.164	−0.010; *p* = 0.102; *q* = 0.136
Base clinical model + SII	0.825	+0.015 (0.001 to 0.029); *p* = 0.036; *q* = 0.072	0.00/0.95	0.163	−0.009; *p* = 0.141; *q* = 0.141
Base clinical model + PINRI	0.834	+0.024 (0.002 to 0.046); *p* = 0.033; *q* = 0.072	−0.02/0.95	0.158	Reference

The base model included age, sex, prestroke mRS, admission NIHSS, eGFR, admission glucose, atrial fibrillation, and reperfusion therapy. C-statistic, Brier score, calibration intercept, and calibration slope were optimism-corrected using 1,000 bootstrap resamples. Delta C values and *p*-values were derived from paired bootstrap comparisons. Extension-versus-base comparisons and, separately, PINRI-versus-CAR, SIRI, PNI, and SII comparisons were FDR-adjusted. LR = likelihood ratio.

**Figure 4:**
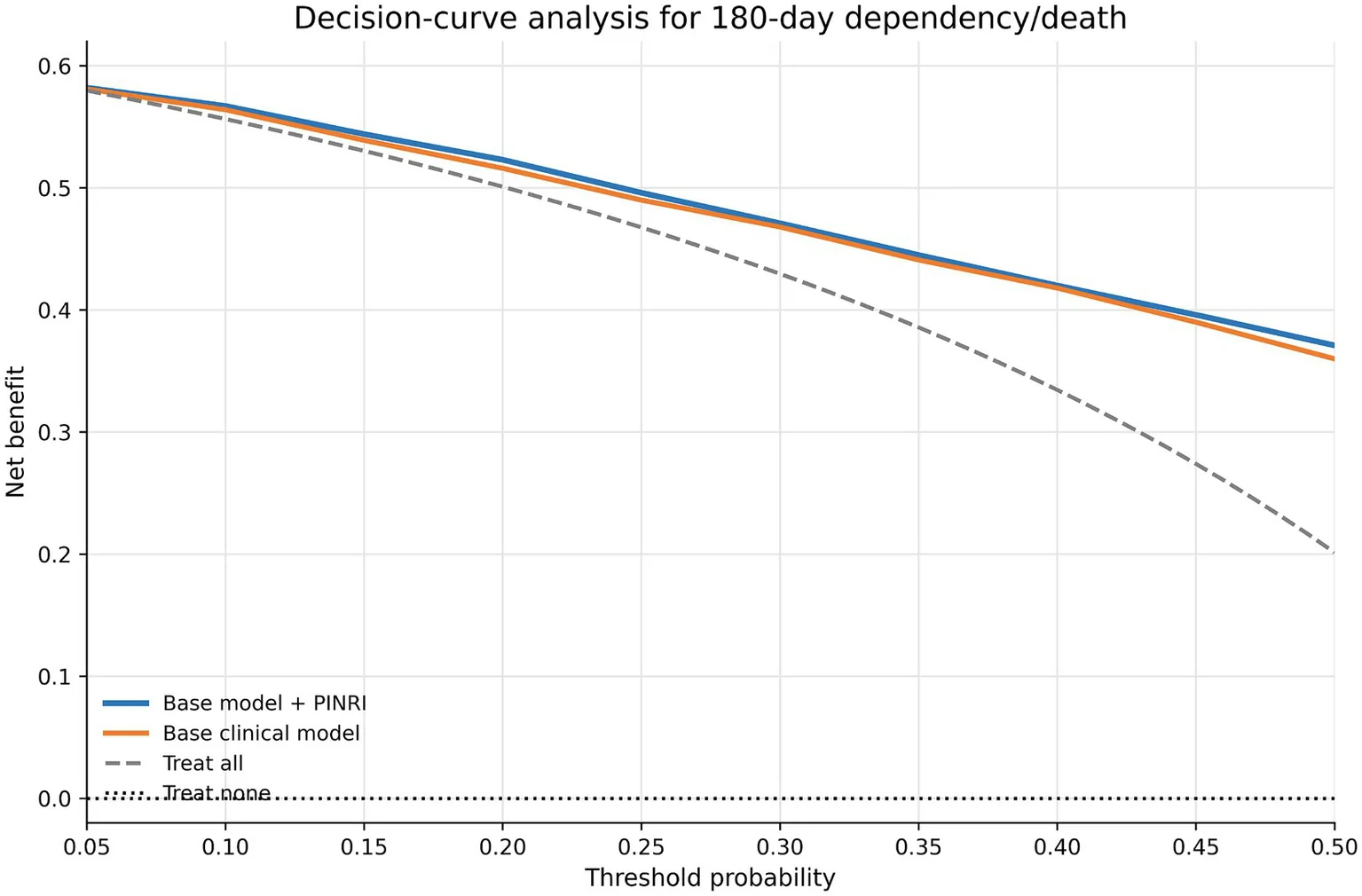
Decision-curve analysis for 180-day dependency or death. Net benefit is plotted across threshold probabilities from 0.05 to 0.50 for the base clinical model, base model plus admission PINRI, treat-all, and treat-none strategies. Curves represent internal evaluation only and do not establish implementation thresholds.

**Figure 5:**
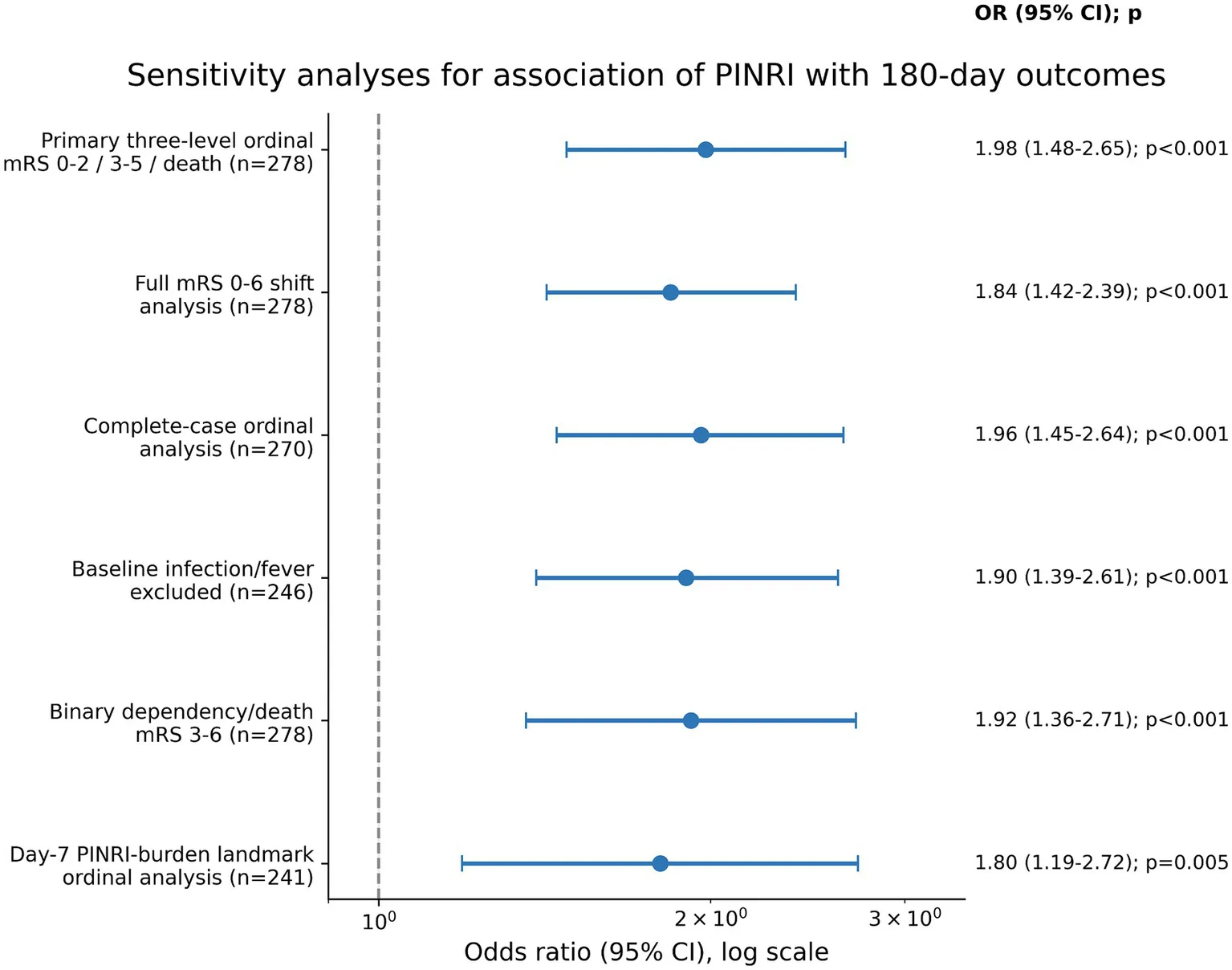
Sensitivity-analysis forest plot. Odds ratios are shown per 1-SD higher admission PINRI or day-7 PINRI burden. Rows differ by analytic population, endpoint, or exposure and should be interpreted as sensitivity checks.

Across otherwise identical biomarker-augmented models, optimism-corrected C-statistics were 0.821 for CAR, 0.822 for SIRI, 0.824 for PNI, 0.825 for SII, and 0.834 for PINRI; corresponding Brier scores were 0.166, 0.165, 0.164, 0.163, and 0.158. PINRI produced the largest numerical improvement over the base model (Delta C, 0.024; 95% CI, 0.002–0.046; bootstrap *p* = 0.033; FDR q = 0.072), but pairwise differences between the PINRI model and component/comparator models were not statistically significant after FDR control (all *q* ≥ 0.100; Table 3). Calibration intercepts ranged from −0.02 to 0.00 and slopes from 0.95 to 0.96. The full mRS 0–6 sensitivity analysis (*n* = 278) was directionally consistent with the primary analysis (adjusted common OR per 1-SD higher PINRI, 1.84; 95% CI, 1.42–2.39; *p* < 0.001), and proportional-odds diagnostics did not indicate an important violation (score-test *p* = 0.27). Figure 5 reports the exact denominator for every sensitivity analysis.

## Discussion

In this single-center cohort of patients aged ≥80 years with AIS, a higher PINRI derived from routine laboratory measurements remained associated with worse 180-day functional survival status after adjustment for age, sex, prestroke mRS, admission NIHSS, atrial fibrillation, renal function, glucose, and reperfusion therapy. The effect size was clinically interpretable: each 1-SD higher admission PINRI was associated with approximately twofold higher adjusted odds of a worse 180-day functional survival state, and the highest tertile had more than threefold higher adjusted odds ratio compared with the lowest tertile. First-week PINRI burden also showed a consistent day-7 landmark association. Importantly, incremental analyses showed a modest increase in internal discrimination and net benefit when PINRI was added to a strong clinical base model. PINRI showed the largest paired C-statistic gain over the base model, but marker-versus-base gains and direct PINRI-versus-marker differences did not remain significant after FDR control; the composite therefore showed numerical, not established, superiority.

Acute ischemia can trigger immune activation, endothelial injury, catabolism, and systemic inflammatory shifts that influence complications and outcome (8, 9). Older stroke patients may also have limited physiological reserve, frailty, dysphagia, and nutritional vulnerability, reducing the tolerance of acute illness and rehabilitation (5, 6, 10). Recent older-adult AIS data show that nutritional indices such as PNI may stratify prognosis, while albumin and CONUT-related evidence support the broader relevance of nutritional status after stroke (11, 26, 27). Because albumin and lymphocyte counts are influenced by multiple acute processes and contribute to more than one component, equal-weight summation may amplify overlapping information rather than represent three distinct biological domains. The index should therefore be read as an early systemic-risk marker rather than the proof that inflammation or nutrition is the sole driver of poor outcome.

This study extends prior biomarker work while also illustrating its limitations. Recent studies support the prognostic relevance of nutritional indices and albumin-based nutritional status after ischemic stroke (11, 26, 27), while stroke-specific CAR evidence links higher C-reactive protein-to-albumin ratio with unfavorable outcomes (12, 13). Meta-analytic evidence also supports associations between systemic immune-inflammation indices, systemic inflammatory response indices, and AIS outcomes (14, 15). The present analysis differs by focusing on patients aged 80 years or older, treating death as the worst functional survival state, and quantifying incremental value beyond a conventional clinical model. This distinction matters because biomarker studies can appear convincing when assessed only by unadjusted group differences, whereas clinical usefulness depends on whether the marker adds interpretable information after NIHSS, prestroke disability, renal function, glucose, and treatment opportunity are considered. The observed gain in discrimination was therefore interpreted as supportive but not transformative. The direct internal comparison showed the largest numerical C-statistic and lowest Brier score for PINRI, but pairwise differences versus CAR, SIRI, PNI, and SII were not statistically significant. These derivation-cohort data therefore support incremental information beyond the clinical base model but do not establish that PINRI is superior to simpler indices.

PINRI uses routine admission tests and can be calculated within a derivation dataset without imaging predictors, omics, or nonstandard monitoring. If externally validated, reported with fixed derivation constants, and locally calibrated, a high PINRI could be tested as a supportive-care trigger for earlier attention to swallowing, infection surveillance, nutrition assessment, rehabilitation readiness, and family counseling. This aligns with evidence that nutritional status scores such as CONUT are associated with functional outcome, complications, and mortality after ischemic stroke (27). It also aligns with rehabilitation literature suggesting that the timing and safety of very early rehabilitation require careful individualization after AIS (28). Importantly, PINRI should not be used in isolation to withhold reperfusion therapy, rehabilitation, or supportive care. Older patients with high systemic vulnerability may be precisely the group needing more structured supportive assessment, not therapeutic nihilism.

The single-center setting limits generalizability, and external validation is essential before clinical implementation. Residual confounding is likely because PINRI may reflect unmeasured frailty, stroke subtype, lesion burden, large-vessel occlusion, socioeconomic factors, care limitations, or preexisting inflammatory disease. Imaging predictors were intentionally excluded to maintain feasibility, limiting comparison with models that include infarct volume, vascular occlusion, or recanalization. Baseline infection/fever and dysphagia were strongly imbalanced across PINRI tertiles and could partly drive CRP, albumin, leukocyte patterns, nutrition, aspiration risk, dependency, and death; infection-excluded analyses were consistent, but reverse causation and mediation cannot be eliminated. The exposure was internally standardized, and fixed z-score constants and externally valid thresholds require validation before use outside the derivation setting. The 7-day onset-to-arrival eligibility window means admission biomarkers may represent different biological phases across patients. The day-7 burden analysis was restricted to survivors with repeated laboratories and may be affected by survival, discharge, and measurement-selection bias. The available sample size was adequate for the primary prognostic-factor analysis but not for definitive interactions, threshold transportability claims, or external validation. These limitations require a cautious interpretation of the observed associations and model-performance gains.

In conclusion, admission PINRI and first-week PINRI burden were associated with 180-day functional-survival outcomes in acute ischemic stroke patients aged 80 years or older. The index offers an internally standardized routine-data composite prognostic marker that complements, but does not replace, age, prestroke function, and NIHSS. The next step is multicenter external validation, reporting of stable derivation constants and thresholds, and prospective testing of whether PINRI-informed supportive-care pathways can improve patient-centered outcomes without restricting access to evidence-based stroke treatment. The index is currently a prognostic and risk-stratification marker, not a validated clinical decision-making threshold.

## Data Availability

The raw data supporting the conclusions of this article will be made available by the authors, without undue reservation.
