# Supplementary material for: A prognostic inflammation-nutrition reserve index for 180-day functional-survival outcomes after acute ischemic stroke in patients aged 80 years or older
Source: Front Neurol. 2026 Oct 23;17:1910335. doi: 10.3389/fneur.2026.1910335 (PMC13739518; doi:10.3389/fneur.2026.1910335)
Supplement: Supplementary file 1 [file Table_1.docx]

**Supplementary Table S1. Cohort-specific centering and scaling constants for reproduction of PINRI**

| **Quantity** | **Transformation** | **Mean** | **SD** | **Standardized expression** |
| --- | --- | --- | --- | --- |
| CAR component | ln(CAR + 0.01) | -1.1157 | 0.8894 | [ln(CAR + 0.01) + 1.1157] / 0.8894 |
| SIRI component | ln(SIRI + 0.01) | 0.7208 | 0.6337 | [ln(SIRI + 0.01) - 0.7208] / 0.6337 |
| PNI component | PNI | 44.8004 | 4.6021 | (PNI - 44.8004) / 4.6021 |
| Equal-weight component sum | zCAR + zSIRI - zPNI | 0.0000 | 2.3126 | (zCAR + zSIRI - zPNI) / 2.3126 |
| Final PINRI | Standardized sum | 0.000 | 1.000 | Final analysis scale |

Reproduction formula: PINRI = {[(ln(CAR + 0.01) + 1.1157)/0.8894] + [(ln(SIRI + 0.01) - 0.7208)/0.6337] - [(PNI - 44.8004)/4.6021]}/2.3126. Natural logarithms and sample SDs (n-1 denominator) were used; the admission-cohort constants were also applied to repeated measurements.
